# Intestinal Absorption of Triterpenoids and Flavonoids from *Glycyrrhizae radix et rhizoma* in the Human Caco-2 Monolayer Cell Model

**DOI:** 10.3390/molecules22101627

**Published:** 2017-09-29

**Authors:** Xiao-Xue Wang, Gui-Yan Liu, Yan-Fang Yang, Xiu-Wen Wu, Wei Xu, Xiu-Wei Yang

**Affiliations:** 1 School of Life Science and Technology, Beijing Institute of Technology, No. 5, Zhongguancun South Street, Haidian District, Beijing 100081, China; 18813007559@163.com; 2 State Key Laboratory of Natural and Biomimetic Drugs, Department of Natural Medicines, School of Pharmaceutical Sciences, Peking University Health Science Center, Peking University, No. 38, Xueyuan Road, Haidian District, Beijing 100191, China; yangyanfang@bjmu.edu.cn (Y.-F.Y.); wuxiuwen0725@126.com (X.-W.W.); high-xu@163.com (W.X.)

**Keywords:** *Glycyrrhizae radix et rhizoma*, triterpenoids, flavonoids, human intestinal Caco-2 cell monolayer model, passive diffusion

## Abstract

*Glycyrrhizae radix et rhizoma* has been used as a traditional Chinese medicine for the treatment of various diseases. Triterpenoids and flavonoids from the plant have many beneficial effects and their chemical structures are modified in the gastrointestinal tract after oral administration. However, absorption of these triterpenoids and flavonoids still needs to be defined. Here, the uptake and transepithelial transport of the selected major triterpenoids, glycyrrhizin (**1**), glycyrrhetic acid-3-*O*-mono-β-d-glucuronide (**2**), and glycyrrhetinic acid (**3**); and the selected major flavonoids, licochalcone A (**4**), licochalcone B (**5**), licochalcone C (**6**), echinatin (**7**), isoliquiritin apioside (**8**), liquiritigenin (**9**), liquiritin apioside (**10**) isolated from *Glycyrrhizae radix et rhizoma*, were investigated in the human intestinal epithelium-like Caco-2 cell monolayer model. Compounds **3**, **5**–**7**, and **9** were designated as well-absorbed compounds, **2** and **4** were designated as moderately absorbed ones, and **1**, **8**, and **10** were assigned for the poorly absorbed ones. The absorption mechanism of well and moderately absorbed compound was mainly passive diffusion to pass through the human intestinal Caco-2 cell monolayer. These findings provided useful information for predicting their oral bioavailability and the clinical application.

## 1. Introduction

**Figure 1:**
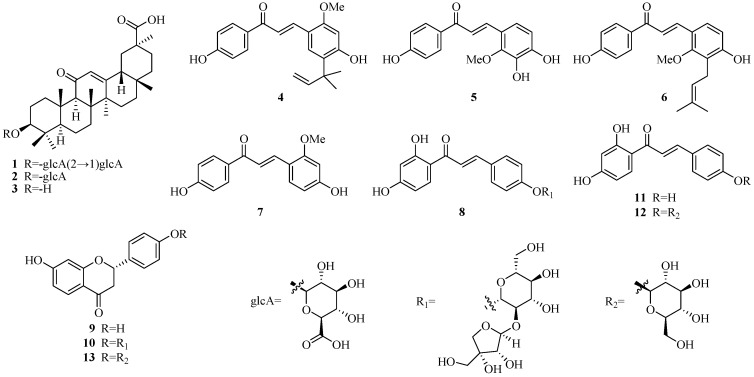
The structures of compounds **1**–**10** isolated from *Glycyrrhizae radix et rhizoma* and three related compounds **11**–**13**.

The genus *Glycyrrhiza* consists of about 30 species with a nearly global distribution, of which about 18 species are found in China. Among them, only three species—named *Glycyrrhiza uralensis* Fisch., *Glycyrrhiza inflata* Bat., and *Glycyrrhiza glabra* L.—have been used as traditional Chinese medicines for the treatment of hepatitis, spasmodic cough, gastric ulcer, etc. [1]. Phytochemical studies showed that over 300 diverse compounds have been identified from the genus *Glycyrrhiza* [2,3,4,5]. Among them, triterpenoids and flavonoids were the two major active substances, exerting a variety of pharmacological activities including antiviral [6,7,8], anti-inflammatory [9,10,11,12], antioxidative [13,14], hepatoprotective [15,16], antimicrobial [17,18,19,20,21,22], antitumor [23,24,25], antiallergic [26,27,28], and potent cytochrome P450 enzyme inhibitory [29] properties. To what extent these components are effective in the human body depends on their bioavailability and metabolism in vivo. As is well-known, intestinal permeability is a crucial factor which influences the bioavailability of drugs, especially for those oral ones. Therefore, rationalizing intestinal permeability of these components is a critical step towards understanding their potential bioactivity. The focus of this study was to investigate the transport and cellular uptake of three major triterpenoids; glycyrrhizin (**1**), glycyrrhetic acid-3-*O*-mono-β-d-glucuronide (**2**) and glycyrrhetinic acid (**3**), and seven flavonoids; licochalcone A (**4**), licochalcone B (**5**), licochalcone C (**6**), echinatin (**7**), isoliquiritin apioside (**8**), liquiritigenin (**9**), and liquiritin apioside (**10**), whose chemical structures are shown in Figure 1, isolated from *Glycyrrhizae radix et rhizoma*, in Caco-2 cell monolayer model [30,31,32], and to predict human intestinal absorption and cellular accumulation of these compounds.

## 2. Results and Discussion

### 2.1. Validation of the Caco-2 Cell Monolayer

The integrity of differentiated Caco-2 cell monolayer was examined by measuring the transepithelial electrical resistance (TEER) with an epithelial voltohmmeter (EVOM, World Precision Instrument, Sarasota, FL, USA). Only cell monolayers with a TEER value above 500 Ω·cm^2^ were used for transport assays [31,32]. The apparent permeability coefficients (*P*_app_) values of propranolol and atenolol, the well- and poor-transported markers by passive diffusion mechanism across Caco-2 cell monolayer, were assessed as (2.51 ± 0.21) × 10^−5^ cm/s and (2.35 ± 0.11) × 10^−^^7^ cm/s, respectively. The results were in a good agreement with the reported values [32], indicating that the applicability of the cell monolayer as in vitro intestinal absorption model was verified. 3-(4,5-Dimethyl-2-thiazolyl)-2,5-diphenyl-2*H*-tetrazolium bromide (MTT) assays [32] showed that all compounds at their maximum test concentration exerted no significant influences on the cell viability.

### 2.2. Validation of High Performance Liquid Chromatography Analysis Method

The high performance liquid chromatography (HPLC) analysis methods for the all test compounds had been validated. The standard calibration curves were constructed by plotting peak area (*y*) versus concentration (*x*, µM). Regression equations, coefficient correlations (*r*^2^), and concentration ranges of the calibration curve for the test compounds were listed in Supplemental Materials (Table S1). The methodological evaluation including the precision, accuracy, and method recovery were summarized in Table S2. The HPLC method was confirmed to be compliant with the Guidance for Industry Bioanalytical Method Validation of FDA [33]. The HPLC method validations of compounds **1**, **8**, and **10** were not performed, because they were extremely difficult to transport through Caco-2 cell monolayer.

### 2.3. Bidirectional Transport Determination

#### 2.3.1. Bidirectional Transport of Triterpenoids **1**–**3**

The bilateral (apical side (AP)→basolateral side (BL) and BL→AP) *P*_app_ values of triterpenoids **1**–**3** are summarized in Table 1. Compound **1** was not detected in the receiving sides after incubation for 90 min in both AP→BL and BL→AP transports, indicating that compound **1** was very poorly absorbed. Whereas the *P*_app_ values of compound **3** were well over 10^−5^ cm/s, which was comparable to that of propranolol ((2.51 ± 0.21) × 10^−5^ cm/s), a well-transported marker of the transcellular pathway [32], so compound **3** was designated as a well-absorbed compound. The *P*_app_ values of compound **2** in the bidirectional transportation were of 1 × 10^−6^ cm/s magnitude, which lay between that of propranolol and atenolol, so compound **2** was designated as a moderately absorbed compound.

Compound **3** is a triterpenoid aglycone, while compounds **1** and **2** are C_3_-*O*-β-d-bisglucopyranuronoside and C_3_-*O*-β-d-monoglucopyranuronoside of **3** (Figure 1), respectively. Apparently, reducing the number of sugar moieties increased the membrane permeability. Compound **1** displayed very poor intestinal absorption that may be attributed to poor membrane permeability. The comparable bidirectional *P*_app_ values (a ratio of 1.06−1.11 for *P*_app BL→AP_/*P*_app AP→BL_) of compounds **2** and **3** suggested passive diffusion as the main transport mechanism. It has been demonstrated in many reports that compound **1** shows substantial pharmacological effects [2,6]. On the other hand, it has also been revealed that compound **1** can be transformed to compounds **2** and **3** by intestinal bacteria following the oral intake of licorice in humans [34]. Therefore, predictions could be made that the compounds **2** and **3** were absorbed in blood as the active metabolite of licorice, especially compound **3**.

#### 2.3.2. The Time Course and Concentration-Dependence of Permeation of Triterpenoids **2** and **3**

**Figure 2:**
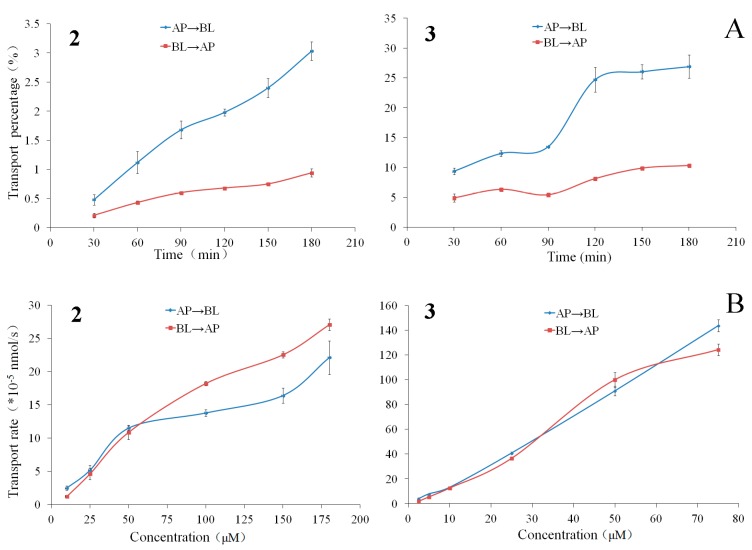
The transport percentage as a function of time at 50 μM (**A**) and transport rates as a function of concentration at 90 min (**B**) of the compounds **2** and **3** in Caco-2 cell monolayer. Data are the mean ± SD (*n* = 3).

**Figure 3:**
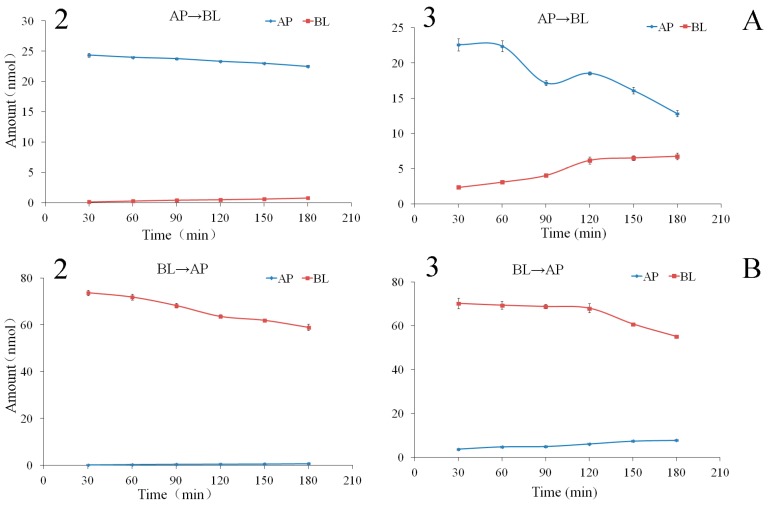
Kinetics curves of the compounds **2** and **3** transports in Caco-2 monolayer from apical to basolateral direction at 50 μM. (**A**) AP→BL, (**B**) BL→AP. Data are the mean ± SD (*n* = 3).

The bilateral permeation of compounds **2** and **3** increased approximately linearly with time (0–180 min) at 50 μM (Figure 2), and the rates of membrane permeation increased approximately linearly at 90 min (Figure 2) within the test range of concentration (10–175 μM for compound **2** and 2.5–75 μM for compound **3**). From kinetic curves presented in Figure 3, the amount of compounds **2** and **3** decreased approximately linearly in AP side and increased approximately linearly in BL side within the incubation time.

#### 2.3.3. Bidirectional Transport of Chalcones **4**–**8**

The bidirectional *P*_app_ values of chalcones **4**–**8** are summarized in Table 1. Chalcones **5**–**7** were designated as well-absorbed compounds, while chalcones **4** and **8** were designated as a moderately and poorly absorbed compounds, respectively. The *P*_app_ values of chalcone **11** (isoliquiritigenin) were reported to be (1.46 ± 0.01) × 10^−5^ cm/s from AP to BL direction and (9.35 ± 0.43) × 10^−6^ cm/s from BL to AP direction [35], and those of chalcone **12** (isoliquiritin) were reported to be (8.69 ± 0.15) × 10^−7^ cm/s from AP to BL direction and (7.68 ± 0.66) × 10^−7^ cm/s from BL to AP direction in our previous papers [36]. The results indicated that chalcone aglycones were well or moderately absorbed compounds. Whereas the magnitude of the bidirectional *P*_app_ values of chalcone glycosides (chalcones **12** and **8**) were 1 × 10^−7^ cm/s or below, they were designated as the poorly absorbed compounds.

#### 2.3.4. The Time Course and Concentration-Dependence of Membrane Permeation of Chalcones **4**–**7**

**Figure 4:**
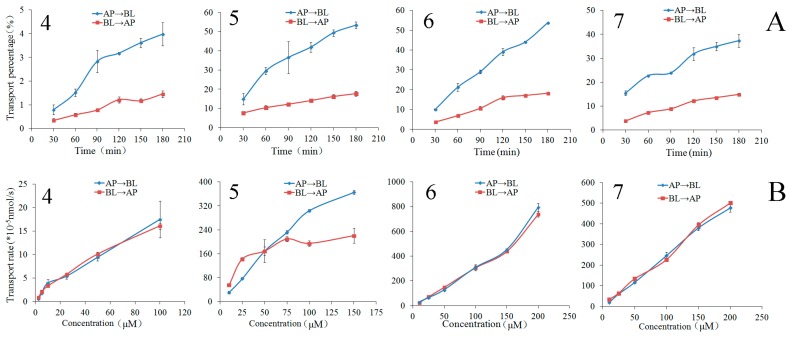
The transport percentage as a function of time at 50 μM (**A**) and transport rates as a function of concentration at 90 min (**B**) of the chalcones **4**–**7** in Caco-2 cell monolayer. Data are the mean ± SD (*n* = 3).

**Figure 5:**
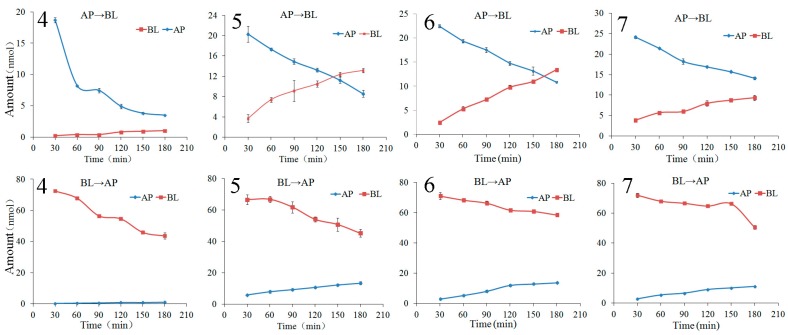
Transport kinetics curves of the chalcones **4**–**7** in Caco-2 monolayer from apical to basolateral direction at 50 μM. Data are the mean ± SD (*n* = 3).

The bilateral permeation of the chalcones **4**–**7** increased approximately linearly with time (0–180 min) at 50 μM (Figure 4), while the rates of membrane permeation increased approximately linearly at 90 min (Figure 4) within the test range of concentration (2.5–100 μM for chalcone **4**, 10–150 μM for chalcone **5**, 10–200 μM for chalcones **6** and **7**). From kinetic curves presented in Figure 5, the amount of the chalcones **4**–**7** decreased in AP side and increased in BL side approximately linearly within the incubation time.

#### 2.3.5. Bidirectional Transport of Flavonones **9** and **10**

The bidirectional *P*_app_ values of flavonones **9** and **10** are summarized in Table 1. Flavonone **9** was assigned as a well-absorbed compound with *P*_app_ value of around 1 × 10^−5^ cm/s. Flavonone **10** was found to hardly permeate Caco-2 cell monolayer with *P*_app_ value < 1 × 10^−7^ cm/s. The *P*_app_ values of flavonone **13** (liquiritin) were reported to be (5.40 ± 0.16) × 10^−7^ cm/s from AP to BL direction and (5.32 ± 0.31) × 10^−7^ cm/s from BL to AP direction in our previous study [36], and it was designated as a poorly absorbed compound. The decreasing order of the permeability was **9** > **13** > **10**, i.e., disaccharide glycoside (**10**) showed less permeability than monosaccharide glycoside (**13**), and both of them had far less permeability than the corresponding aglycone (**9**). This was in accordance with the finding in the case of triterpenoids **1**–**3** that the permeability of compounds in the Caco-2 cell monolayer can be reduced with the increase in the number of glycosyl groups on the core molecular nuclei.

#### 2.3.6. The Time Course and Concentration-Dependence of Permeation of Flavonone **9**

**Figure 6:**
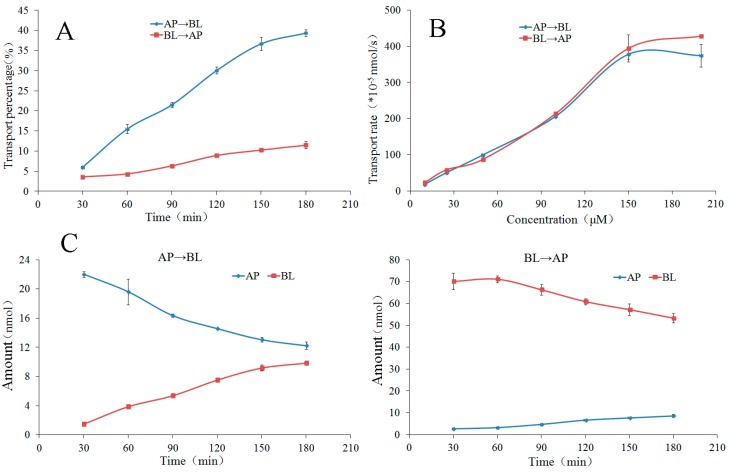
The transport percentage as a function of time at 50 μM (**A**), transport rate as a function of concentration at 90 min (**B**) and transport kinetics curves (**C**) of the flavonone **9** in Caco-2 monolayer. Data are the mean ± SD (*n* = 3).

The bilateral permeation of the flavonone **9** increased approximately linearly with time (0–180 min) at 50 μM (Figure 6), while the rates of permeation increased approximately linearly at 90 min (Figure 6) within the test range of concentration (10–200 μM). From kinetic curves presented in Figure 6, the amount of the flavonone **9** in AP side decreased approximately linearly and in BL side increased approximately linearly within the incubation time.

At the end of the transport experiments, the mass balance was calculated (see Supplementary data, Table S3). The recoveries of compounds **2**, **3**, **5**–**7**, and **9** were 85.06–98.24% in all bidirectional transport studies with very low cell accumulation. This suggests no significant first-pass metabolism during their intestinal absorption and transport. The recovery of compound **4** was relatively lower in the AP→BL transport with the higher intracellular uptake in all bidirectional transport. The efflux ratios (Table 1) of the compounds **2**–**7**, and **9** were within the range of 0.88–1.22, suggesting that their bidirectional transport was comparable and lack of directional preference. The results of the concentration-dependency presented in Figure 2, Figure 4 and Figure 6 suggested a passive diffusion mechanism of compounds **2**–**7**, and **9** across the Caco-2 cell monolayer. The kinetics curves for time-dependency (Figure 2, Figure 4 and Figure 6) indicated no efflux or active transport and again confirmed the passive diffusion mechanism. In general, the passively transported compounds with *P*_app AP→BL_ values > 1 × 10^−6^ cm/s can cross the intestinal barrier efficiently with the percentage of absorption > 20% [32]. *P*_app AP→BL_ values of compounds **2**–**7**, and **9** all exceeded 1 × 10^−6^, predicting that they would be absorbed in human intestine.

**Figure 7:**
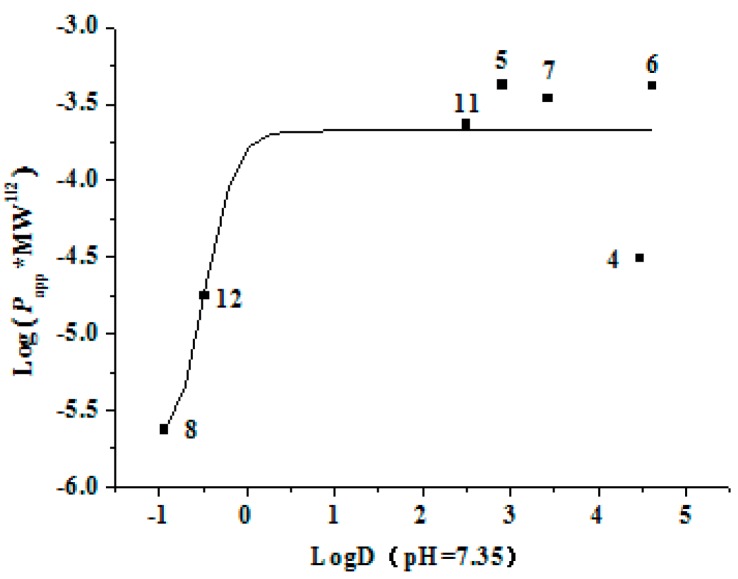
The relationship between log (*P*_app AP→BL_ × MW^1/2^) and log D (pH = 7.35) for seven chalcones (**4**–**8**, **11**, and **12**).

Physicochemical characters such as log D (logarithm of octanol–water partition coefficient) and MW (molecular weight) were generally utilized for the prediction of the permeability of compounds [37]. An analysis of the relationship between the log D, MW, and the determined *P*_app_ value of different kinds of compounds may help to rich the predictive database. The log D values at pH 7.35 of compounds **1**–**13**, calculated with Pallas 3.3.2.6 ADME/Tox Software (CompuDrug, Bal Harbor, FL, USA), as well as their MW values were shown in Table 1. Herein, a sigmoid trendline of log (*P*_app AP→BL_ × MW^1/2^) versus logD was plotted (Figure 7) with Origin Pro 7.5 SR1 (Origin Lab. Corporation, Northampton, MA, USA) to elucidate the structure-permeability relationship of chalcones **4**–**8**, **11**, and **12**. It was found that the permeability of chalcones increased in the log D range of −1–3, which was consistent with the common regulation that the more lipophilicity one compound shows, the more permeability it has; however, those of chalcones kept nearly invariable in the log D range of 3−5, which led to a higher permeability with the exception of chalcones **4**.

## 3. Experimental Section

### 3.1. Assayed Compounds

Triterpenoids glycyrrhizin (**1**), glycyrrhetic acid-3-*O*-mono-β-d-glucuronide (**2**), and glycyrrhetinic acid (**3**) were supplied by Natural Product Sample Library in State Key Laboratory of Natural and Biomimetic Drugs of Peking University (Beijing, China). Chalcones licochalcone A (**4**), licochalcone B (**5**), licochalcone C (**6**), and echinatin (**7**) were obtained from Tianjin Chroma-standard Medical Science & Technology Development Co. Ltd. (Tianjin, China), and isoliquiritin apioside (**8**) was obtained from Shanghai Tauto Biotech Co. Ltd. (Shanghai, China). Flavonones liquiritigenin (**9**) and liquiritin apioside (**10**) were obtained from Shanghai Tauto Biotech Co. Ltd. (Shanghai, China).

### 3.2. Chemicals and Reagents

HPLC grade methanol (MeOH) was purchased from Fisher Scientific (Fair lawn, NJ, USA). Analytical grade acetic acid and Hank’s Balanced Salts Solution (HBSS) were purchased from Beijing Chemical Works (Beijing, China). Water was purified by a Mili-system (Millipore, Bedford, MA, USA). Propranolol, atenolol and dimethyl sulfoxide (DMSO) were purchased from Sigma-Aldrich Co. (St. Louis, MO, USA). Penicillin-streptomycin solution was obtained from Suolaibao Technology Ltd. (Beijing, China). Dulbecco's Modified Eagle's Medium (DMEM), fetal bovine serum (FBS), phosphate buffered saline (PBS), nonessential amino acids (NEAA), and trypsin were supplied by Gibco^®^ Laboratories (Life Technologies Inc., Grand Island, NY, USA).

### 3.3. Instrumental Analyses

Quantitative analysis of compounds **2**–**7** and **9** was performed on a Dionex Ultimate™ 3000 UHPLC system (Dionex Corp., Sunnyvale, CA, USA), comprised of Ultimate 3000 pump, autosampler, column compartment, and diode array detector. The signals were acquired and processed applying a Chromeleon version 6.80 software (Dionex Corp., Sunnyvale, CA, USA). HPLC separation was performed on a Dikma Diamonsil™ C_18_ column (250 mm × 4.6 mm, 5 μm, Dikma Technology, Inc., Beijing, China). The mobile phase was consisted of MeOH and 0.6% acetic acid in aqueous solution (*v*/*v*) in 81:19 for compound **2**, 86:14 for compound **3**, 70:30 for compound **4**, 45:55 for compounds **5**, **6**, and **9**, 50:50 for compound **7**, with a flow rate of 1 mL/min, a column temperature of 30 °C and a injected volume of 10 μL for compounds **2**–**7** and **9**. The detection wavelength was set at 254 nm for compounds **2** and **3**, 372 nm for compound **4**, 360 nm for compound **5**, 276 nm for **6**, 370 nm for **7**, and 276 nm for compound **9**. The calibration curves were constructed by plotting peak area (*y*) versus concentration (*x*, µM), which concentration series (µM) were 5, 25, 50, 75, 150, 200 for compound **2**; 2.5, 5, 10, 15, 25, 50 for compound **3**; 5, 10, 15, 25, 50, 75 for compound **4**; 2.5, 5, 10, 25, 50, 75 for compound **5**; 5, 10, 25, 50, 75, 150 for compound **6**; 10, 25, 50, 75, 150, 200 for compound **7**; 5, 10, 25, 75, 100, 200 for compound **9**. The amounts of compounds **1**, **8**, and **10** in the receiving chambers were below the lower limit of detection (LLOD). Quantification was carried out by peak area measurements in comparison with the calibration curves. Methodology was examined for precision, accuracy, and recovery (see Supplemental Material Table S1) and was demonstrated to meet the requirements of determination.

### 3.4. Cell Culture

The human intestinal Caco-2 cell line (ATCC #HTB-37) was purchased from American Type Culture Collection (ATCC, Rockville, MD, USA). The cell culture was carried out in a Sanyo MCO-15 AC carbon dioxide (CO_2_) incubator (Sanyo Electric Co., Ltd., Osaka, Japan). The integrity of the Caco-2 cell monolayer was examined by measuring the TEER with an epithelial voltohmmeter (EVOM, World Precision Instrument, Sarasota, FL, USA) [32]. All cells used in this study were between passages 30 and 40.

### 3.5. Caco-2 Cell Permeability

The Caco-2 cell monolayer permeability was carried out according to the previously reported method [32,38]. Briefly, the Caco-2 cells were maintained in DMEM containing 10% FBS, 1% NEAA, 100 units/mL of penicillin, and 100 µg/mL of streptomycin, in a constant humidity atmosphere of 5% CO_2_ and 95% air at 37 °C. For confluence and differentiation, cells were seeded at a density of 1 × 10^5^ cells/cm^2^ into 12-well Transwell plates (insert diameter 12 mm, pore size 3.0 μm, membrane growth area 1.12 cm^2^, Costar^®^ #3402) and were allowed to grow for 21 days before the permeation experiment. On day 21, the monolayers with TEER values > 500 Ω·cm^2^ were qualified for the transport experiment. The transport study was initiated by the careful removal of the culture medium from AP and BL sides of the inserts. Caco-2 monolayers were rinsed twice with pre-warmed HBSS and were incubated by pre-warmed HBSS for 30 min at 37 °C. Stock solutions of assayed compounds **1**–**10** were prepared in DMSO and diluted to 50 µM with HBSS. The final DMSO concentration was less than 2%, a concentration that did not alter the Caco-2 cells viability or permeability. The assayed solutions (50 µM) were added to the AP side (0.5 mL, for absorption transport) or BL side (1.5 mL, for efflux transport) of the inserts, while the receiving chamber contained the corresponding volume of HBSS. Incubation was performed at 37 °C for 90 min, with shaking at 50 rpm. Samples were collected from the inserts and lyophilized. The dried sample was then sonicated with the appropriate volume of MeOH, followed by centrifugation at 15,000× *g* for 10 min. The supernatant was filtered through a 0.45 μm filter and injected into HPLC system for quantitative analysis.

The AP to BL or BL to AP permeability coefficient (*P*_app_) value of each compound was calculated based on the following equation:

*P*_app_ = (dQ/dt) × (1/A) × (1/C_0_) (cm/s) (1)

where dQ/dt is the rate of the appearance of the test compound on the receiver compartment (μmol/s), C_0_ is the initial test compound concentration on the donor compartment (μmol/mL), and A is the surface area of Caco-2 monolayer (cm^2^).

### 3.6. Time- and Concentration-Dependent Transport of the Compounds ***2**–**7*** and ***9*** across the Caco-2 Cell Monolayer

To observe the time-dependence, 50 μM of the compounds **2**–**7** and **9** were added to either AP or BL side of the inserts. While shaking the samples (37 °C, 50 rpm), 1.3 mL aliquots were taken from BL side or 0.45 mL aliquots were taken from AP side at 30 min intervals from 30 to 180 min.

To observe the concentration-dependence, the compounds **2**–**7** and **9** were added to either AP or BL side of the inserts at the final concentration in the range of 10–175 μM for compound **2**, 2.5–75 μM for compound **3**, 2.5–100 μM for compound **4**, 10–150 μM for compound **5**, 10–200 μM for compounds **6**, **7**, and **9**. After shaking the samples (37 °C, 50 rpm) in a shaking water bath for 90 min, aliquots were collected as described above. As in the case of time-dependent transport experiments, the kinetics curves of the compounds **2**–**7** and **9** were drawn at an initial concentration of 50 μM and intervals from 30 to 180 min.

### 3.7. Intracellular Accumulation and Recovery

To measure the amount of intracellular accumulation, the Caco-2 cells were rinsed three times with ice-cold HBSS at the end of transport experiments. After three freeze (−20 °C)-thaw (room temperature) cycles, the lysed Caco-2 cells were lyophilized. The dried cells were sonicated in 0.2 mL of 70% aqueous MeOH for 20 min and then centrifuged at 15,000× *g* for 10 min. The supernatant was filtered through a 0.45 μm filter and a 10 μL aliquot was injected into the HPLC system for analysis. To check the mass balance, the recoveries of the assayed compounds were measured at both sides of the insert and intracellular accumulation of the Caco-2 cells.

## 4. Conclusions

The transport rates of the six selected compounds **3**, **5**–**7**, and **9**, lipophilic aglycones including major triterpenoid, chalcone, and flavonone compounds in licorice, increased linearly with the concentration and saturation was not observed at the concentrations tested, indicating that the permeation mechanism for these compounds was passive diffusion. The magnitude of the bidirectional flux of these compounds was comparable to propranolol (*P*_app_ = (2.51 ± 0.21) × 10^−5^ cm/s). These data suggest that the compounds **3**, **5**–**7**, and **9** were well-absorbed compounds and were transported mainly through passive diffusion by the transcellular pathway. The recoveries of compounds **3**, **5**–**7**, and **9** were higher in bidirectional transport studies with very low cell accumulation, suggesting metabolism may not be involved during their intestinal absorption and transport. In spite of the fact that chalcone **4** was also an aglycone, the higher accumulation in Caco-2 cells resulted in its *P*_app_ value laying between *P*_app_ values of propranolol and atenolol, and chalcone **4** was designated as a moderately absorbed compound. Permeation of the bisglycosides **1**, **8**, and **10**, across the Caco-2 cell monolayer was not detected, suggesting their *P*_app_ values less than 1 × 10^−7^ cm/s, and they were designated as the very poorly absorbed compounds. While the monoglycosides **2** with 1 × 10^−6^ cm/s degree of *P*_app_ value, **12** and **13** with 1 × 10^−7^ cm/s degree of *P*_app_ value [36] were designated as the moderately or poorly absorbed compounds, indicating that the number of sugar groups linked in aglycone molecule was one of the important factors to affect their intestinal absorption by comparison with the corresponding aglycone. In addition, analysis on the relationship between the log D, MW, and the determined *P*_app_ value of different kinds of compounds may help to rich the predictive database of drug ADME/T. These new findings provided useful information for predicting their oral bioavailability, pharmacokinetics, and the clinical application as well as determination of the bioactive substance basis of *Glycyrrhizae radix et rhizoma* [39,40].

## Figures and Tables

**Table 1:** The bidirectional *P*_app_ values of compounds **1**–**13** in Caco-2 cell monolayer (*n* = 3). ^a^

Analytes	*P*_app AP→BL_^b^ (×10^−6^ cm/s)	*P*_app BL→AP_^c^ (×10^−6^ cm/s)	Efflux Ratio ^d^	MW	Log D (pH = 7.35)
**1**	0	0	–	822	−8.02
**2**	1.55 ± 0.09	1.65 ± 0.95	1.06	646	−2.94
**3**	12.63 ± 0.40	14.00 ± 1.30	1.11	470	2.57
**4**	1.68 ± 0.13	1.78 ± 0.03	1.06	338	4.48
**5**	24.71 ± 2.80	30.18 ± 2.40	1.22	286	2.91
**6**	22.51 ± 1.00	26.44 ± 1.30	1.17	338	4.62
**7**	20.70 ± 1.00	23.80 ± 0.30	1.15	270	3.43
**8**	0	0	–	550	−0.94
**9**	17.80 ± 0.60	15.68 ± 0.30	0.88	256	2.95
**10**	0	0	–	550	−0.23
**11**	14.60 ± 0.10	9.35 ± 0.43	0.64	256	2.50
**12**	0.87 ± 0.02	0.77 ± 0.07	0.89	418	−0.48
**13**	0.54 ± 0.02	0.53 ± 0.03	0.98	418	0.02

^a^ The incubation time was up to 90 min. ^b^ Transport of assayed compounds from AP to BL direction. ^c^ Transport of assayed compounds from BL to AP direction. ^d^ The ratio of *P*_app BL→AP_ to *P*_app AP→BL_.

## Supplementary Materials

Supplementary materials are available online: Table S1 for regression data of compounds **2**–**7** and **9**. Table S2 for precision, accuracy and recovery of compounds **2**–**7** and **9**. Table S3 for the total recovery and the intracellular accumulation percentage of the assayed compounds **2**–**7** and **9** from the Caco-2 cell monolayer at the end of transport.
