# Supplementary material for: Intestinal Absorption of Triterpenoids and Flavonoids from Glycyrrhizae radix et rhizoma in the Human Caco-2 Monolayer Cell Model
Source: Molecules. 2017 Sep 29;22(10):1627. doi: 10.3390/molecules22101627 (PMC6151599; doi:10.3390/molecules22101627)
Supplement: Supplementary file 1 [file molecules-22-01627-s001.pdf]

Supplementary Material:

Intestinal Absorption of Triterpenoids and flavonoids from *Glycyrrhizae radix et rhizoma* in the Human Caco-2 Monolayer Cell Model

Xiao-Xue Wang, Gui-Yan Liu, Yan-Fang Yang, Xiu-Wen Wu, Wei Xu, Xiu-Wei Yang

Table S1. Regression data of compounds **2–7** and **9**.

Analytes	Standard curve	R^2	Linear range (μM)
**2**	$y = 16.5348x - 93.6358$	0.9980	5–200
**3**	$y = 30.360x - 25.134$	0.9990	2.5–50
**4**	$y = 0.6355x + 0.1744$	0.9990	5–75
**5**	$y = 20.4584x + 16.6306$	0.9990	2.5–75
**6**	$y = 7.19718x + 21.3983$	0.9980	5–150
**7**	$y = 19.6885x - 10.9668$	0.9990	10–200
**9**	$y = 56.2833x + 30.0455$	0.9990	5–200

Table S2. Precision, accuracy and recovery of compounds **2**–**7** and **9**.

Analytes	QC	Precision (RSD %)		Accuracy		Method recovery (%)	
	μM	Intra-day	Inter-day	Intra-day	Inter-day	Mean	RSD
**2**	10	0.8	3.75	105.23	103.78	95.27	2.67
	75	0.45	2.55	100.77	98.27	96.04	2.06
	150	1.53	2.09	106.59	109.36	92.78	1.56
**3**	5	1.16	3.66	98.37	95.67	93.17	2.06
	30	0.28	0.31	106.22	108.72	105.6	1.99
	60	0.09	0.82	99.1	96.38	93.19	2.5
**4**	10	0.4	1.49	105.23	103.78	95.27	2.67
	50	0.1	1.24	100.77	98.27	96.04	2.06
	80	0.16	1.34	106.59	109.36	92.78	1.56
**5**	10	1.16	3.66	98.37	95.67	93.17	2.06
	60	0.28	0.31	106.22	108.72	105.65	1.99
	120	0.09	0.82	99.1	96.38	93.19	2.5
**6**	10	0.72	1.87	99.45	110.1	98.24	1.39
	80	0.74	1.95	104.7	103.76	89.99	2.05
	160	0.42	1.75	95.34	105.43	99.4	1.94
**7**	10	1.52	3.92	95.77	98.29	106.22	2.15
	80	0.57	4.25	102.35	105.56	110.23	1.06
	160	0.26	2.81	97.38	100.2	107.11	1.85
**9**	10	0.08	1.33	99.27	103.82	97.21	1.88
	80	0.88	1.13	95.27	98.56	98.51	1.23
	160	0.28	1.03	103.67	106.21	94.31	0.97

Table S3. The total recovery and the intracellular accumulation percentage of the assayed compounds **2**–**7** and **9** from the Caco-2 cell monolayer at the end of transport (*n* = 3).

Analytes	AP→BL		BL→AP	
	**Total recovery/%[a]**	**CA/%[b]**	**Total recovery/%[a]**	**CA/%[b]**
**2**	97.04 ± 0.36	–	91.63 ± 1.27	–
**3**	85.06 ± 2.33	0.25 ± 0.01	98.24 ± 0.59	–
**4**	81.88 ± 0.67	50.52 ± 1.88	90.36 ± 2.74	42.56 ± 2.36
**5**	96.2 ± 0.19	–	94.53 ± 2.89	–
**6**	97.52 ± 1.26	–	96.13 ± 4.55	–
**7**	96.88 ± 2.67	–	97.71 ± 3.29	–
**9**	87 ± 2.78	–	94.79 ± 4.29	–

[a] Total recovery: the percentage of the total amount of assayed compound recovered from both sides and intracellular accumulation in Caco-2 cell monolayer relative to the original amount; [b] CA (cell accumulation): the percentage of assayed compound that accumulated in the cell monolayer relative to the original amount.
–: not detected.
